# Comparison of Framingham Risk Scores (FRS), Joint British Society (JBS3), and American College of Cardiology/American Heart Association (ACC/AHA) Cardiovascular Risk Scores Among Adults With First Myocardial Infarction

**DOI:** 10.7759/cureus.33221

**Published:** 2023-01-01

**Authors:** Madhu P Raj, Vishwanath Krishnamurthy, Eilene Basu, Vijay Balaji, Varun Vinayak Prakash Rao, Nithin R

**Affiliations:** 1 Internal Medicine, Ramaiah Medical College, Bangalore, IND

**Keywords:** prevention of mi, smoking, india, cardiovascular risk scores, first myocardial infarction

## Abstract

Introduction: The prevalence of myocardial infarction (MI) among young Indian adults is on the rise with reports suggesting 32.7% of all deaths in men and 32.6% of all deaths in women between 2010-13 were due to cardiovascular diseases (CVDs). Though various long-term cohort studies have established risk assessment scores none of them are specific to the Indian population. In this study, we look to establish which scoring system among the American College of Cardiology (ACC), Joint British Society (JBS3) and Framingham Risk Scores (FRS) would be reliable for the Indian population. A timely intervention based on the most reliable score can help mitigate cardiovascular diseases.

Materials and methods: In this cross-sectional study, we included Indian adults, aged more than 40 years, with first MI. Patients previously on lipid lowering drugs were excluded. Demographic data, history, clinical information, laboratory data and other investigations were noted. Subsequently the predicted cardiovascular risk scores based on JBS3, ACC, and FRS were calculated and divided into low risk, intermediate and high risk based on the categorization of the risk scores individually.

Results: There were 102 (79.1%) males and 23 (17.8%) females with a mean age of 51.01 years (standard deviation [SD]=12.82, p value <0.001). There was considerable prevalence of type 2 diabetes mellitus with 56 (47.1%) of the subjects being known diabetics.

The mean 10-year risk of MI based on ACC was 12.42% (SD=10.45), mean JBS3 score was 14.45% (SD=12.67) and mean FRS score was 15.75% (SD=14.71). FRS scores when categorized, 48 (40.3%) patients had low risk, 30 (23.3%) had medium risk and 43 (33.3%) had high risk. As for ACC score, 39 (35.8%) patients were in low risk and 29 (26.6%) in intermediate risk, borderline in 18 (16.5%) and high risk in 23 (21.1%). In JBS3 scores, 53 (46.5%) patients were in low risk, 32 (28.1%) were in moderate risk and 29 (25.4%) in high risk.

Conclusion: The absolute value of 10-year risk scores was highest for FRS scores. The proportion of patients whose scores were under the category of high risk was highest for FRS.

## Introduction

As a developing nation, India is facing an epidemiological transition in the pattern of diseases. The major burden of diseases have shifted from communicable to non-communicable diseases [1], with the obvious exception of COVID-19. With an increasing burden of myocardial infarction (MI) cases which are at par with the developed countries, it becomes necessary to strengthen preventive strategies. More and more young adults are being hit with MI in India. As per reports, cardiovascular diseases (CVDs) led to 32.7% of all deaths in men and 32.6% of all deaths in women between 2010-13 [2]. Various long-term cohort studies have established risk factors and risk assessment scoring systems. However, the majority of these scoring systems do not necessarily consider ethnicity and are not specific to the Indian population. In this study, we retrospectively analyze which scoring system among the American College of Cardiology (ACC), Joint British Society-3 (JBS3) and Framingham Risk Score (FRS) is the most reliable for the Indian population since these three risk scores are the most commonly used worldwide. A timely intervention based on the most reliable score can help mitigate cardiovascular diseases and suppress the epidemiological transition.

## Materials and methods

This is a cross sectional study with a retrospective method of data collection. The subjects included in this study were Indian adults, aged above 40 years, with first MI admitted in a tertiary medical college hospital. The patients who were previously on medications that alter the blood lipid profile, such as statins, were excluded. A study carried out by Bansal et al. [3]^ ^has shown the precision in terms of estimating high cardiovascular risk of different assessment models to be 13.5% (FRS), 20.2% (JBS3), 55.9% (ACC), 38.3% (World Health Organization). Based on the above findings of the study, to exhibit statistical significance, we included a minimum of 125 subjects for the study with 95% confidence, with 7% precision.

Data were collected after obtaining written informed consent. Demographic data, history of presentation and risk factors were noted. Vital parameters such as heart rate, blood pressure, and oxygen saturation (SpO2) were measured. Clinical examination findings, electrocardiogram findings and 2D echocardiography (ECHO) findings were noted too. Diagnosis of MI was defined based on the 4th Universal definition of MI [4]. Angiographic findings of patients with MI were noted. The following laboratory values were also taken into account: complete blood counts (CBC), serum electrolytes, fasting lipid profile (FLP), renal function tests (RFT), liver function tests (LFT), glycated hemoglobin (HbA1C), cardiac troponins, and fasting blood sugar (FBS). Based on the data collected above, the predicted cardiovascular risk scores based on JBS3, ACC, and FRS were calculated and divided into low risk, intermediate and high risk based on the categorization of the risk scores individually.

Quantitative continuous variables such as age, body mass index (BMI), systolic blood pressure (SBP), diastolic blood pressure (DBP), low-density lipoprotein (LDL) cholesterol, etc were expressed in terms of mean and standard deviation (SD). Categorical variables like presence of hypertension, diabetes mellitus, smoking habits, etc were expressed as frequency and percentages. Ten-year cardiovascular risk score was calculated and patients were categorized into various levels such as mild, moderate and high risk. Differences in the proportion of high-risk groups were tested for statistical significance among the different risk assessment models by the Chi-Square test. Differences in the mean values were tested for statistical significance by Student-t test. Correlation coefficient was calculated between the different cardiovascular risk assessment models.

## Results

The subjects in this study were predominantly males (102 [79.1%]) as compared to 23 (17.8%) females. Type 2 diabetes mellitus was present in 56 (47.1%) cases. Nine subjects (7.6%) had a previous family history of MI. Seventy-five (58.1%) patients were active smokers while 38 (31.9%) patients admitted having smoked previously but have abstained from smoking now. Thirty-five (29.9%) patients were on antihypertensive medication. Other comorbidities seen were as follows: history of atrial fibrillation in 14 (10.9%) patients, chronic kidney disease (CKD) in three (2.6%) patients and five (4.3%) patients were known cases of rheumatoid arthritis. These variables are tabulated in Table 1.

**Table 1:** Descriptive statistics on categorical variables

Demographic Parameters	frequency	percentage
Male sex	102	79.1
Female sex	23	17.8
Diabetes present	56	47.1
Family History present	9	7.6
History of Smoking	38	31.9
Currently Smoking	75	58.1
Anti hypertensive treatment	35	29.9
History of atrial fibrillation	14	10.9
Chronic renal disease	3	2.6
Rheumatoid arthritis	5	4.3

The mean age of the subjects was 51.01 years (SD=12.82 years). The variation in the ages was significant among the patients with a p value <0.001. The pulse rate on an average was 87 beats per minute (SD=18.74) with average BP of systolic 131 mmHg (SD=26.01) and diastolic 80 mmHg (SD=14.58) which surprisingly falls in the bracket of normal range despite hypertension being an established risk factor. The mean arterial pressure on average was 94 mmHg (SD=20.55) and respiratory rate was 19 cycles/minute (SD=2.65). These statistics however were significant when tested with a p value <0.001. The above-mentioned statistics are tabulated in Table 2. 

**Table 2:** Descriptive statistics of clinical parameters

	Mean	SD	P Value
Age (years)	51.01	12.82	<0.001
Pulse rate (beats per minute)	87	18.74	<0.001
Systolic blood pressure (mmHg)	131	26.01	<0.001
Diastolic blood pressure (mmHg)	80	14.58	<0.001
Mean arterial pressure (mmHg)	94	20.55	<0.001
Respiratory rate (cycles/minute)	19	2.65	<0.001
Blood oxygen saturation (%)	92	7.78	0.038

Mean values of lipid profiles were as follows: total cholesterol levels were 170.79 mg/dL (SD=52.49). Triglycerides were 169.50 mg/dL (SD=99.66). Mean HDL cholesterol was 37.65 (SD=10.26) while non-HDL cholesterol was 128.88 mg/dL (SD=54.15) and LDL cholesterol was 104.02 mg/dL (SD=48.58). Mean of total cholesterol to HDL ratio was 4.84 (SD=2.09). The components of the lipid profile among all the subjects were tested to be statistically significant (p-value<0.001). Mean values of random blood sugar (RBS) were 214 mg/dL (SD=76.39). This however when tested for statistical significance was found to be not significant (p-value=0.40). This data on lipid profiles are represented in Table 3.

**Table 3:** Descriptive statistics of laboratory parameters HDL: high-density lipoprotein, LDL: low-density lipoprotein, TC/HDL: total cholesterol/high-density lipoprotein ratio, mg/dL: milligrams per deciliter

Lab parameters	Mean	SD	P value
Total cholesterol (mg/dL)	170.79	52.49	<0.001
HDL cholesterol (mg/dL)	37.65	10.26	<0.001
Non-HDL cholesterol (mg/dL)	128.88	54.15	<0.001
LDL cholesterol (mg/dL)	104.02	48.58	<0.001
Triglycerides (mg/dL)	169.50	99.66	<0.001
TC/HDL ratio	4.84	2.09	<0.001
Random blood sugar (mg/dL)	214.00	76.39	0.40

**Figure 1:**
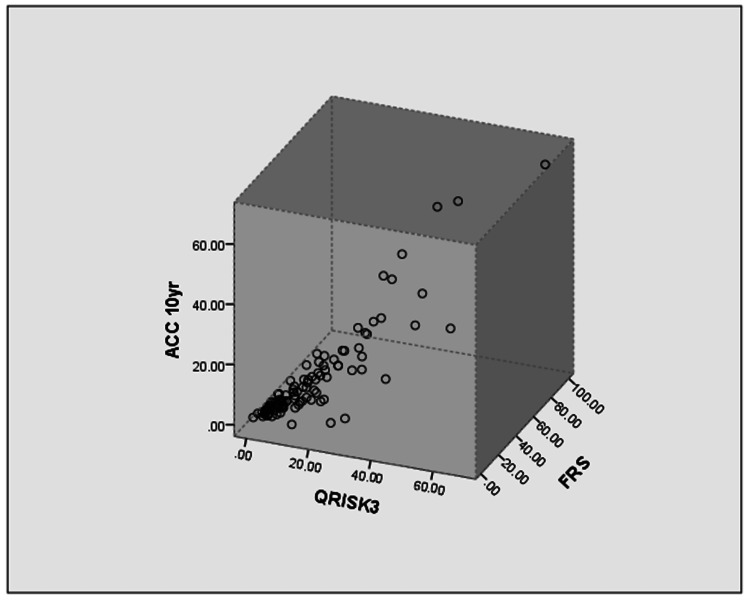
3D scatter plot of ACC vs FRS vs JBS3 Note: ACC 10 yr refers to the risk of an adverse cardiovascular event in the next 10 years Note: QRISK3 indicates the JBS3 scores as it refers to the software used by the JBS for calculation of risk scores ACC: American College of Cardiology, JBS3: Joint British Society, FRS: Framingham Risk Scores

**Figure 2:**
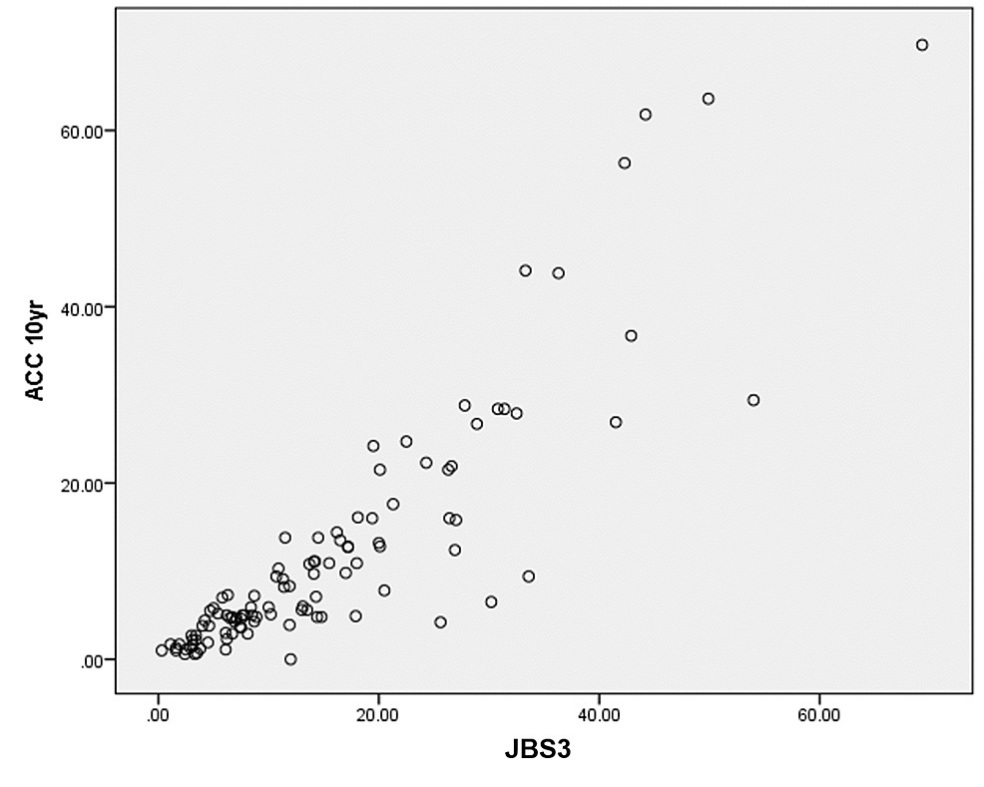
Plot of ACC 10-year risk score versus JBS3 risk score ACC: American College of Cardiology, JBS3: Joint British Society

**Figure 3:**
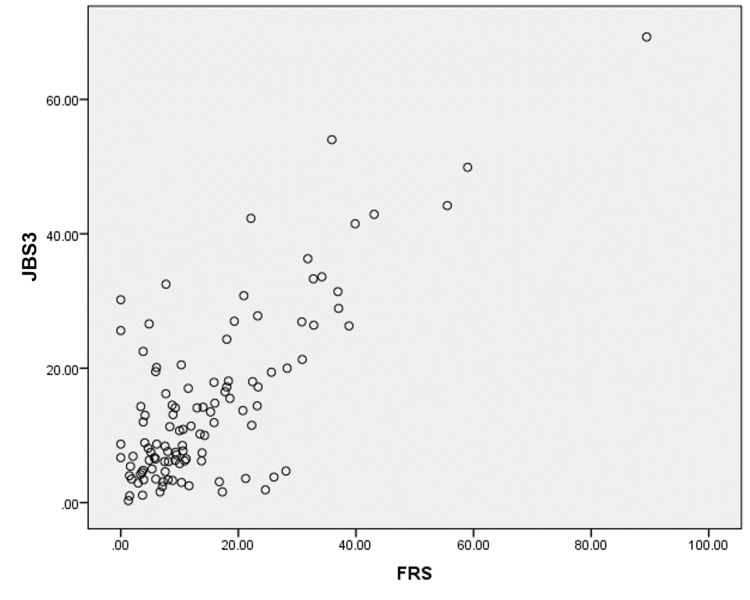
Plot of JBS3 versus FRS risk scores JBS3: Joint British Society, FRS: Framingham Risk Scores

Risk scores were calculated using standardized calculators officially published by the respective institutions. Patients’ average ACC score was 12.42% (SD=10.45), mean JBS3 score was 14.45% (SD=12.67), and mean FRS score was 15.75% (SD=14.71). The difference in the mean value of various risk scores was not statistically significant. FRS score was calculated in all 125 patients, 48 (40.3%) patients were in the low-risk category, 30 (23.3%) were in medium-risk and 43 (33.3%) were in the high-risk category. As for ACC score, 39 (35.8%) patients were in low-risk and 29 (26.6%) in intermediate-risk, borderline in 18 (16.5%) and high-risk in 23 (21.1%). In JBS3 scores, 53 (46.5%) patients were in low-risk, 32 (28.1%) were in moderate-risk and 29 (25.4%) in high-risk (Tables 4, 5). The relation of the three scores was assessed using Pearson correlation (r) (Table 6). ACC and JBS3 had a correlation of r=0.887. The correlation between JBS3 with FRS and ACC with FRS was found to be r=0.761. The correlation of each score has been represented as graphs in Figures 1, 2, 3.

**Table 4:** Descriptive statistics of risk scores ACC: American College of Cardiology, JBS3: Joint British Society, FRS: Framingham Risk Scores

Risk scores	Mean	SD
ACC (%)	12.42	10.45
JBS3 (%)	14.54	12.67
FRS (%)	15.75	14.71

**Table 5:** Frequencies of risk strata of FRS, ACC, JBS3 ACC: American College of Cardiology, JBS3: Joint British Society, FRS: Framingham Risk Scores

RISK SCORES	ACC	JBS3	FRS
	Frequency (%)	Frequency (%)	Frequency (%)
LOW RISK	39 (35.8%)	53(46.5%)	48 (40.3%)
MEDIUM	-	32 (28.1%)	30 (23.3%)
INTERMEDIATE	29 (26.6%)	-	-
BORDERLINE	18(16.5%)	-	-
HIGH RISK	23 (21.1%)	29 (25.4%)	43 (33.3%)

**Table 6:** Pearson correlation between the various risk scores ACC: American College of Cardiology, JBS3: Joint British Society, FRS: Framingham Risk Scores

Correlations	ACC 10yr	JBS3	FRS
ACC 10yr	1	0.887	0.761
JBS3	0.887	1	0.761
FRS	0.761	0.761	1

In Figure 1, we see that each point represents the various risk scores of a given patient. The scatter plot being almost planar indicates the similarity between the various risk scores. However, the Pearson correlation quantifies the linearity. Figure 2 shows the relation between ACC 10-year risk and JBS3 score, though the plot shows a somewhat linear relationship there is a skew towards the right indicating higher JBS3 scores for most patients. Similarly in Figure 3, though somewhat linear we can see the skew towards JBS3 scores as compared to FRS scores. However the stratification elucidates the precision of FRS over the other scoring systems. 

## Discussion

Cardiovascular diseases are relatively more aggressive and tend to develop at an earlier age, as young as 14 years [4]. As demonstrated in our study, MI without prodromal symptoms is more prevalent in younger individuals with CAD [5]. According to histological studies, these plaques include more lipids and have less cellular scar tissue than plaques found in older patients, giving us a clue on lesser chronicity and rapid progression. With a higher propensity of dislodgement of these plaques, ST-elevation MI at a younger age is statistically more common than chronic stable angina [6].

Early intervention and mitigation of the ensuing problems can prevent both morbidity and mortality associated with cardiovascular events. Several tools have been developed as a result of extensive research studies spanning decades. These risk score systems, when inculcated in a specific algorithm and executed, show the individual’s risk of developing an adverse cardiovascular event in the future. The result, expressed in percentage, indicates the likeliness or statistical probability of developing a cardiac event. Despite a large portion of subjects from the cardiovascular study being from a diverse population, most risk assessment tools do not consider ethnicity as a variable in their algorithm. Moreover with a significantly higher prevalence of certain non-communicable diseases and lifestyle disorders among Indians [7,8] and the implication of genetic diversity as a contributory factor for the same, the question arises if the tools can be used in our setup of the Indian population without modifications to the algorithm. 

There have been attempts at different approaches for cardiovascular risk assessment among Indians. For example, recalibrating the FRS by multiplying the calculated FRS by an appropriate correction factor of 0.27 and even offering a specific chart for Indians that can be more accurate for our population (e.g. in RISK WHO), the predictive accuracy of the contemporary risk models has not been adequately evaluated in Indians. The JBS3 score, which has been confirmed in ethnic Indians, is actually more prevalent among non-resident Indians. This study attempted to examine these several risk indicators in order to determine which of them has the best predictive value for identifying high-risk individuals. There were 149 patients without prior cardiac illnesses who presented with their first acute MI (mean age 59.4±10.6 years; 123 [82.6%] men).

The FRS, WHO risk prediction charts, ACC pooled cohort equations and JBS3 were used to estimate what would have been their predicted 10-year risk of cardiovascular events if these patients had presented just prior to suffering an acute MI. The results of the present study showed that the FRS cardiovascular risk assessment tool could identify the maximum number of patients as high risk for future cardiovascular events. The results are similar to a study done by Garg et al. [9]. However, in a study done by Paul et al. [10] on 579 Kerala-based subjects aged between 40 and 60 years (mean age of 49.9±5.7) showed that JBS3 was a better model for cardiovascular risk assessment in Kerala-based Indian sub-ethnic population. Similar results were found in a study done by Bansal et al. [3]. While there are studies that have concluded that FRS is a better assessment tool for the Indian population, we also have studies that say JBS3 is a better risk assessment tool. In our study we have seen better results with FRS as a risk assessment tool. The thoroughness of examination and data collection in our study substantiates the results.

In terms of limitations of the study, substantial evidence derived from such studies most often require a long-term cohort study with prospective data collection and follow up. However in our study we have tried our best to eliminate this factor. Demography and lifestyle before and after the acute cardiac event do not differ. Since parameters such as lipid profile do not change over a course of a few hours, the values before and after a cardiac event would be the same. Other variables such as vital parameters are expected to be deranged in a majority of the patients but the margin of error is comparable in all patients. Hence the bias gets nullified here and the level of precision is high. We have hence actively overcome the statistical bias except for the sample size and have arrived at a valuable conclusion regarding which risk score is more relevant for our contemporary Indian population. However, our study is not a population-based study and further studies with larger sample sizes are the need of the hour to be able to initiate primordial prevention of cardiovascular disease. 

## Conclusions

Males with diabetes who have a history of smoking or are currently smoking and are on treatment for hypertension have the highest risk of developing MI. The absolute values of 10-year risk scores were the highest for FRS scores. The proportion of patients whose scores clearly indicated high risk was highest for FRS. Though these results when compared to ACC and JBS3 were not statistically significant, it is important to take these into absolute consideration. Further, the implication of this is that patients presenting to the OPD with cardiovascular symptoms or are known diabetics, hypertensives or patients with dyslipidemia can be assessed using FRS i.e implementation at ground level. Despite apparently normal clinico-laboratory parameters, in case of high-risk scores the patient can be initiated on appropriate means of primordial prevention of MI which may include lifestyle modifications with or without pharmacological agents.

## References

[1] Yadav S, Arokiasamy P (2014). Understanding epidemiological transition in India. Glob Health Action.

[2] (2013). Causes of Deaths in India, 2010-2013. https://censusindia.net/.

[3] Bansal M, Kasliwal RR, Trehan N (2014). Comparative accuracy of different risk scores in assessing cardiovascular risk in Indians: a study in patients with first myocardial infarction. Indian Heart J.

[4] Thygesen K, Alpert JS, Jaffe AS, Chaitman BR, Bax JJ, Morrow DA, White HD (2018). Fourth universal definition of myocardial infarction (2018). Circulation.

[5] Enas EA, Yusuf S, Mehta J (1996). Meeting of the International Working Group on Coronary Artery Disease in South Asians. 24 March 1996, Orlando, Florida, USA. Indian Heart J.

[6] Glover MU, Kuber MT, Warren SE, Vieweg WV (1982). Myocardial infarction before age 36: risk factor and arteriographic analysis. Am J Cardiol.

[7] Sinha SK, Krishna V, Thakur R (2017). Acute myocardial infarction in very young adults: a clinical presentation, risk factors, hospital outcome index, and their angiographic characteristics in North India-AMIYA Study. ARYA Atheroscler.

[8] Sharma M, Majumdar PK (2009). Occupational lifestyle diseases: an emerging issue. Indian J Occup Environ Med.

[9] Garg N, Muduli SK, Kapoor A, Tewari S, Kumar S, Khanna R, Goel PK (2017). Comparison of different cardiovascular risk score calculators for cardiovascular risk prediction and guideline recommended statin uses. Indian Heart J.

[10] Paul P, George N, Shan BP (2020). Cardiovascular risk prediction using JBS3 tool: a Kerala based study. Curr Med Imaging.

